# Nonlocality-Enabled Magnetic Free Optical Isolation in Hyperbolic Metamaterials

**DOI:** 10.3390/ma14112865

**Published:** 2021-05-27

**Authors:** Bartosz Janaszek, Marcin Kieliszczyk, Paweł Szczepański

**Affiliations:** 1 Institute of Microelectronics and Optoelectronics, Warsaw University of Technology, Koszykowa 75, 00-665 Warsaw, Poland; marcin.kieliszczyk@pw.edu.pl (M.K.); pawel.szczepanski@pw.edu.pl (P.S.); 2 National Institute of Telecommunications, 1 Szachowa Str., 04-894 Warsaw, Poland

**Keywords:** hyperbolic metamaterials, spatial dispersion, topological engineering

## Abstract

Hereby, we present an optical isolator (optical diode) based on a hyperbolic metamaterial (HMM). We demonstrate that a grating-free planar linear non-magnetic HMM structure deposited on a high-index substrate, which, due to presence of strong spatial dispersion (non-locality), reveals asymmetrical transmittance and reflectance characteristics for light of arbitrary polarization within a wide angular and spectral range. The presented device may be efficiently utilized to completely block backward and enforce unidirectional propagation in free space and integrated systems without the use of magnetooptical or non-linear effects.

## 1. Introduction

By definition [1], a “true” optical isolator (optical diode) is a device equivalent, in terms of functionality, to an electronic diode, which blocks or diverts all possible states of radiation for backward propagation. This type of device is often used to prevent undesired back propagation that may be harmful to optical instruments and components. In addition, an optical diode may be employed to suppress unwanted interference and interaction between different optical components, as well as to eliminate parasitic light routing in waveguiding systems. Since the first successful realization of optical isolation [2], the most common approach to induce non-reciprocity in an optical system is to employ Faraday effect [3,4,5,6,7,8,9]. However, systems utilizing magneto-optic effect are typically characterized by a large size and cannot be easily implemented in on-chip optical systems [10]. With the development of integrated optics, there exists an ever-growing demand for compatible solutions for optical isolation. Over the last two decades, non-linear optical effects have been considered as promising means for optical isolation in integrated systems [11,12,13,14]. However, optical isolators based on non-linear materials are applicable only to high intensity signals, which often does not eliminate unwanted feedback to a sufficient degree [15]. Recent advances in nanofabrication allowed to achieve new means for non-reciprocity via use of novel photonic components, such as magneto-optical photonic crystals [16,17] or a two-cavity optomechanical system [18]. Moreover, there have been few successful attempts to obtain asymmetrical transmission based on a coupled multiple-microcavity system with balanced gain and loss, i.e., parity-time symmetry [19,20]. Most recent scientific efforts in this field have been oriented towards exploitation of artificially-created structures, the so-called metamaterials [21,22,23,24,25]. Until now, it has been demonstrated that a complex triple-helix metamaterial structure may provide magnetic-free optical isolation within a broad spectral range [25]. What is more, it has been shown that appropriate nanopatterning [23] or presence of magneto-optical response [22] may also lead to broadband asymmetric light transport in a metasurface [22,23]. Recently, hyperbolic metamaterials (HMM) have emerged as a promising class of structures that may be employed to obtain optical-diode behavior [26,27]. It has been demonstrated that asymmetrical transmission of TM-polarized light can be achieved through fabrication of nanopatterned asymmetric metallic grating [27] or magnetization [26].

In this work, we demonstrate that asymmetrical transmission for light of arbitrary polarization may be obtained in a grating-free multilayer planar structure based on non-magnetic linear materials, which provides means for diversion of backward propagation without use of external magnetic field. The proposed optical diode consists of an HMM structure with appropriately designed unit cell enhancing spatial dispersion deposited on a high-index transparent optical substrate [28]. In this work, we demonstrate for the first time an optical isolator that may be realized in a simple and feasible grating-free planar geometry without use of magnetic-optical or non-linear effects. It is also worthwhile to underline that the obtained performance is not a consequence of any unique properties of considered materials, and, thus, may be replicated for other material compositions, i.e., different dielectric and plasmonic material. The working principle of the device is based on strong wavevector dependence of topological phase of iso-frequency dispersion contour of the HMM medium. Thus, depending on the refractive index of incident medium, i.e., air or high-index substrate, as well as the angle of incidence, incoming radiation may “encounter” different type of dispersion of the HMM medium leading to the asymmetrical transmission characteristics of the device. For the purpose of our analysis, we employed effective medium theory (EMT) for acquiring effective permittivity tensor of the HMM medium, as well as transfer matrix method (TMM) for calculation of transmittance and reflectance characteristics. The obtained results indicate that the proposed device reveals optical isolation for waves of arbitrary polarization over wide angular and spectral range.

## 2. Theory

For clarity, we present theoretical foundations of, including effective medium and transfer matrix formalisms, as well as assumptions that were employed in our analysis.

### 2.1. Effective Medium Theory

**Figure 1:**
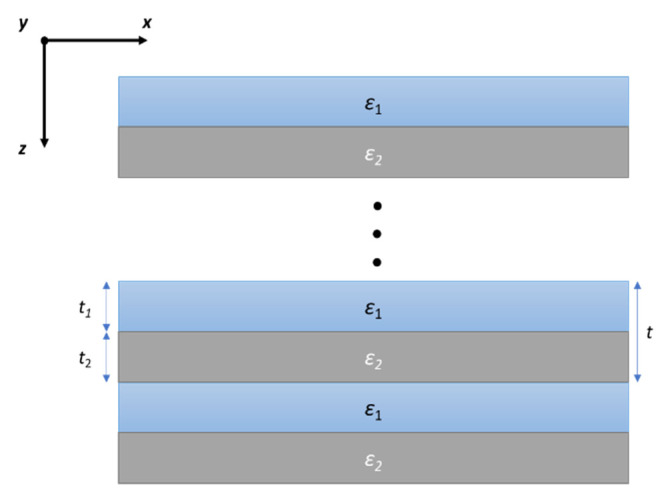
Schematic of a multilayer HMM structure.

According to the local effective medium theory, a periodic multilayer structure composed of two different materials, see Figure 1, may be treated as a uniform anisotropic medium with uniaxial diagonal permittivity tensor with components of the following form [29]:

(1) $$\varepsilon_{xx}=\varepsilon_{yy}=\varepsilon_{\left|\right|}=\frac{t_{1}\varepsilon_{1}(\omega )+t_{2}\varepsilon_{2}(\omega )}{t_{1}+t_{2}}$$

(2) $$\varepsilon_{zz}=\varepsilon_{⊥}=\frac{\varepsilon_{1}(\omega )\varepsilon_{2}(\omega )\left(t_{1}+t_{2}\right)}{t_{1}\varepsilon_{2}(\omega )+t_{2}\varepsilon_{1}(\omega )}$$

where *ε*_1,2_ and *t*_1,2_ correspond to permittivities and layer thicknesses of the materials constituting the unit cell of considered structure, see Figure 1. The local approximation for a multilayer structure is correct as long as the wavelength of radiation *λ* is much longer than the characteristic dimension of the considered structure, i.e., *t/λ*→0 [30]. In this case, *t* = *t*_1_ + *t*_2_ is the thickness of the unit cell.

However, in the case of hyperbolic metamaterial composed of dielectric and plasmonic material, the influence of non-locality can be substantially enhanced by applying the appropriate geometry of the unit cell [28]. This effect can be related to degeneration of plasmon modes arising from coupling between surface waves existing at interfaces between dielectric and plasmonic material [31].

Thus, in our analysis we use a non-local EMT formalism proposed by Chern, which allows to describe structures that do not fulfill the local approximation condition *t/λ*→0 [32]. As a consequence of strong spatial dispersion, a multilayer non-local nanostructure is described as a biaxial anisotropic medium having frequency- and wavevector-dependent permittivity tensor components:

(3) $$\varepsilon_{xx}^{nloc}=\frac{\varepsilon_{\left|\right|}-\frac{\alpha }{12}k_{0}^{2}t^{2}}{1-\frac{1}{12}k_{z}^{2}t^{2}},$$

(4) $$\varepsilon_{yy}^{nloc}=\varepsilon_{\left|\right|}\left(1+\frac{1}{6}k_{x}^{2}t^{2}\right)+\frac{t^{2}}{12k_{0}^{2}}\left(k_{z}^{4}-k_{x}^{4}\right)-\frac{\alpha }{12}k_{0}^{2}t^{2},$$

(5) $$\varepsilon_{zz}^{nloc}=\frac{\varepsilon_{⊥}-\frac{\alpha }{12}k_{0}^{2}t^{2}}{1+\frac{\varepsilon_{⊥}}{\varepsilon_{\left|\right|}}\left(\frac{\beta }{12}k_{x}^{2}t^{2}-\frac{\gamma }{6}k_{0}^{2}t^{2}\right)},$$

Within our analysis that *x-z* plane is the plane of incidence; see Figure 1, thus the wavevector $\vec{k}$ have only *k*_*x*_, *k*_*z*_ non-zero components. The coefficients *α*, *β*, and *γ* in Equations (3)–(5) takes following form:

(6) $$\alpha =\left[f_{1}^{2}\varepsilon_{1}(\omega )+\left(1-f_{1}^{2}\right)\varepsilon_{2}(\omega )\right]\left[\left(1-f_{2}^{2}\right)\varepsilon_{1}(\omega )+f_{2}^{2}\varepsilon_{2}(\omega )\right],$$

(7) $$\beta =\frac{1}{\varepsilon_{1}(\omega )\varepsilon_{2}(\omega )}\left[\left(1-2f_{1}f_{2}\right)\varepsilon_{1}(\omega )+2f_{1}f_{2}\varepsilon_{2}(\omega )\right]\left[2f_{1}f_{2}\varepsilon_{1}(\omega )+\left(1-2f_{1}f_{2}\right)\varepsilon_{2}(\omega )\right],$$

(8) $$\begin{array}{c} \gamma =\frac{1}{\varepsilon_{1}(\omega )\varepsilon_{2}(\omega )}[f_{1}^{3}f_{2}\varepsilon_{1}^{3}(\omega )+f_{1}\left(1-2f_{1}^{2}f_{2}+f_{2}^{3}\right)\varepsilon_{1}^{2}(\omega )\varepsilon_{2}(\omega )\\ +f_{2}\left(1-f_{1}f_{2}^{2}+f_{1}^{3}\right)\varepsilon_{1}(\omega )\varepsilon_{2}^{2}(\omega )+f_{1}f_{2}^{3}\varepsilon_{2}^{3}(\omega )], \end{array}$$

where $f_{i}=t_{i}/t$ is the filling factor of *i-th* layer, where i∈{1, 2}.

### 2.2. Transfer Matrix Method

In this section, we present a transfer matrix method (TMM) formalism allowing for calculating transmittance and reflectance of a system composed of anisotropic media described with a diagonal biaxial permittivity tensor $\bar{\bar{\varepsilon }}=diag([\begin{array}{ccc} \varepsilon_{xx}, & \varepsilon_{yy}, & \varepsilon_{zz} \end{array}])$. In general, such a biaxial medium supports propagation of two orthogonally polarized plane waves, i.e., a transverse electric (TE) wave with field components *E*_y_ and *H*_x_, and a transverse magnetic (TM) wave with field components *H*_y_ and *E*_x_. For the purpose of our analysis, we assume the time dependence *exp* (–*jωt*). Additionally, we limit our investigate to structures uniform in the *x-y* directions. Now, knowing that the plane of incidence is the *x–z* plane, spatial differentiation may be simplified as follows $\begin{array}{cc} \frac{\partial }{\partial x}=jk_{x}, & \frac{\partial }{\partial y}=0 \end{array}$. Based on above assumptions and Maxwell equations [33], the problem of wave propagation through an anisotropic structure may be formulated as the following matrix equation:

(9) $$\frac{\partial \psi (z^{\prime })}{\partial z^{\prime }}-\Omega \psi (z^{\prime })=0$$

where *z’ = z/k_o_* is normalized position, while the characteristic matrix Ω and field vector *ψ* may be described in the following form:

(10) $$\Omega =\left[\begin{array}{cccc} 0 & 0 & 0 & 1-\frac{{\bar{k_{x}}}^{2}}{\varepsilon_{zz}}\\ 0 & 0 & 1 & 0\\ 0 & \varepsilon_{yy}-{\bar{k_{x}}}^{2} & 0 & 0\\ -\varepsilon_{xx} & 0 & 0 & 0 \end{array}\right]$$

(11) $$\psi (z^{\prime })=\left[\begin{array}{c} E_{x}(z^{\prime })\\ E_{y}(z^{\prime })\\ \bar{H_{x}}(z^{\prime })\\ \bar{H_{y}}(z^{\prime }) \end{array}\right]$$

For simplicity of interpretation, we assume normalization of wavevector $\bar{k_{x}}=k_{x}/k_{0}$ as well as magnetic field $\bar{H_{x}}=-j\sqrt{\mu_{0}/\varepsilon_{0}}H_{x}$ and $\bar{H_{y}}=-j\sqrt{\mu_{0}/\varepsilon_{0}}H_{y}$ components. Solution of the Equation (9) may be written as $\psi (z^{\prime })=e^{\Omega z^{\prime }}\psi (0),$ which can be also formulated as an eigenvalue problem [33]:

(12) $$\psi (z^{\prime })=We^{\lambda z^{\prime }}c,$$

where *c* = *W*^−1^*ψ*(0) is a vector of field amplitudes, while *W* and *λ* are eigen-vector and eigen-value matrices of characteristic matrix Ω. By applying continuity condition for field components at the interfaces of the anisotropic layer $\psi_{in}=\psi_{layer}(0),\psi_{layer}(k_{0}L)=\psi_{out}$ to the Equation (11), we can formulate a matrix equation describing the relationship between field amplitudes at reflection ($c_{in}=W_{in}^{-1}\psi_{in}$) and transmission ($c_{out}=W_{out}^{-1}\psi_{out}$) sides of the structure:

(13) $$c_{out}=W_{out}^{-1}W_{layer}e^{\lambda_{layer}k_{0}L}W_{layer}^{-1}W_{in}\cdot c_{in}=T_{layer}\cdot c_{in},$$

where *L* is the thickness of the anisotropic layer and *W*_out_ and *W_in_* are eigen-vector matrices of media surrounding the layer. The complete transfer matrix for a system composed of a single anisotropic layer embedded between semi-infinite media takes the following form:

(14) $$T=\left[\begin{array}{cccc} t_{11} & t_{12} & t_{13} & t_{14}\\ t_{21} & t_{22} & t_{23} & t_{24}\\ t_{31} & t_{32} & t_{33} & t_{34}\\ t_{41} & t_{42} & t_{43} & t_{44} \end{array}\right]=W_{out}^{-1}\cdot T_{layer}\cdot W_{in}.$$

The intensity reflection coefficients (further regarded as reflectance) for TE and TM polarization may be formulated as follows [33]:

(15) $$R_{TE}={\left|\frac{t_{21}t_{33}-t_{23}t_{31}}{t_{11}t_{33}-t_{13}t_{31}}\right|}^{2},$$

(16) $$R_{TM}={\left|\frac{t_{21}t_{43}-t_{41}t_{13}}{t_{11}t_{33}-t_{13}t_{31}}\right|}^{2}$$

In the case of low-loss materials, we can assume that intensity transmission coefficients (further regarded as transmittance) may be estimated as follows:

(17) $$T_{TE}\approx 1-R_{TE},$$

(18) $$T_{TM}\approx 1-R_{TM}.$$

The expressions for reflectance and transmittance of an anisotropic structure, see Equations (17) and (18) will be further employed in our analysis. To calculate characteristic matrix Ω, see Equation (10), the local/nonlocal effective permittivity tensor components of the considered HMM structure will be utilized, see Equations (1)–(5).

## 3. Results

**Figure 2:**
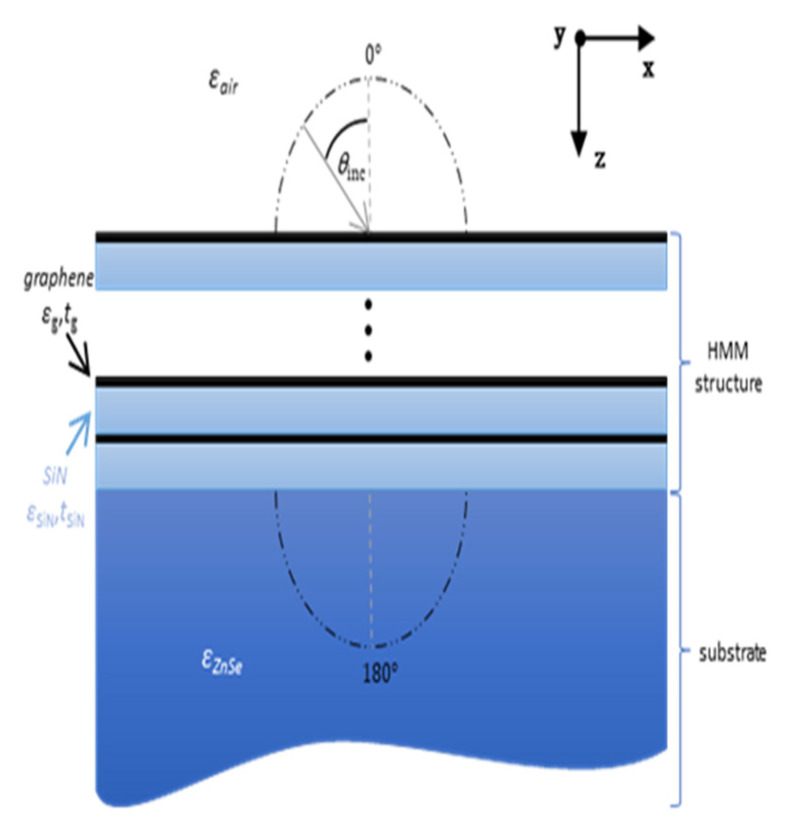
Schematic of the proposed optical diode (optical isolator).

Without losing the generality of considerations, we assume that the unit cell of the considered HMM structure is composed of a monolayer graphene, characterized by permittivity *ε*_1_ = *ε*_g_(*ω*) described by the well-known Kubo formula and thickness *t*_1_ = *t*_g_ = 0.35 nm [34], and a *t*_2_ = 150 nm layer of silicon nitride (SiN) with permittivity *ε*_2_ = *ε*_SiN_(*ω*) calculated via Sellmeier formula [35], compare Figure 1 and Figure 2. It is worth noting that, the assumed unit cell violates the local approximation condition, i.e., *t/λ*→0, within the visible and near-infrared spectral range. Thus, it can be expected that the influence of the spatial dispersion will be substantial [28]. At this point, we would like to underline that the observed nonlocal effects do not originate from any unique properties of chosen materials, which are assumed to be non-magnetic, linear, and local, but rather from interactions between plasmonic modes propagating in the considered multilayer structure [31]. Thus, similar effects may be obtained for different material compositions, i.e., various sets of dielectric and plasmonic material, as indicated in our previous studies [28,33].

The thickness of the HMM structure has been chosen as 1.5 μm, which corresponds to 10 unit cells, a number of unit cells satisfying validity of EMT approach for planar nanostructures [36]. Moreover, we assume that the HMM structure is deposited on a high-index ZnSe substrate *ε*_out_ = *ε*_ZnSe_ ≈ 6.25 [37], while the superstrate is formed of air *ε*_in_ = *ε*_air_ ≈ 1, see Figure 2. The air superstrate and ZnSe substrate, have been assumed as semi-infinite, which is correct approximation as long as coherence length of light is shorter than the path that light travels [38].

Throughout the analysis, the HMM structure is effectively described as an anisotropic biaxial medium via EMT approach, see Equations (3)–(5) where *ε*_1_ = *ε*_g_(*ω*) and *ε*_2_ = *ε*_SiN_(*ω*), while the transmittance and reflectance characteristics are acquired with the help of a transfer matrix method. To determine directional properties of transmission and reflection, including forward and backward propagation, we have considered angles of incidence ranging from 0° to 360°, where 0° indicates normal incidence from the air side, while the 180° is related to the normal incidence from the ZnSe substrate side, see Figure 2. It is worthwhile to underline that all materials constituting the considered system may be considered as low-loss, nonmagnetic, linear, and local.

**Figure 3:**
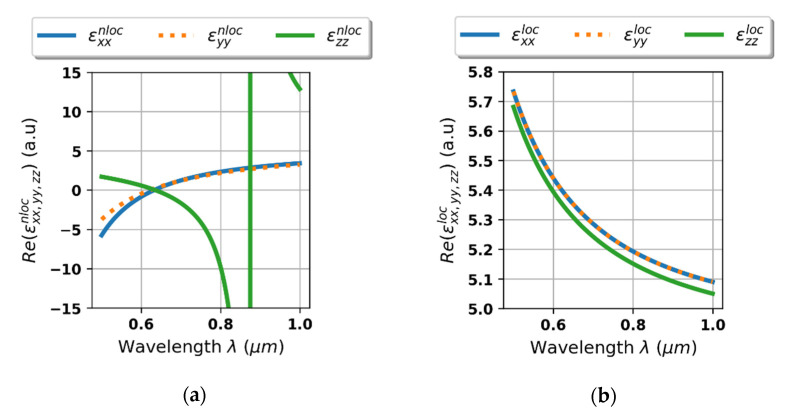
Real parts of effective permittivity tensor components of the HMM structure plotted vs. wavelength, calculated with non-local (**a**) and local (**b**) effective medium approach.

Firstly, to determine the influence of non-locality, we consider spectral characteristics of effective permittivity tensor components of the HMM structure acquired with the help of non-local and local effective medium approach, see Figure 3a,b, respectively. Due to the strong non-locality, the HMM structure reveals Type II hyperbolic dispersion and a strong resonant transition, which cannot be predicted with the help of local EMT approximation [28], compare Figure 3a,b.

**Figure 4:**
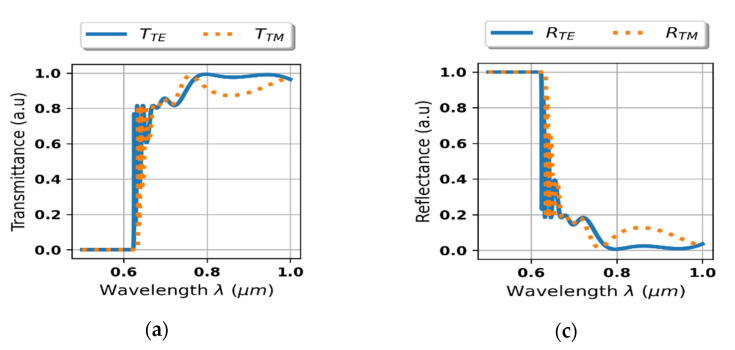

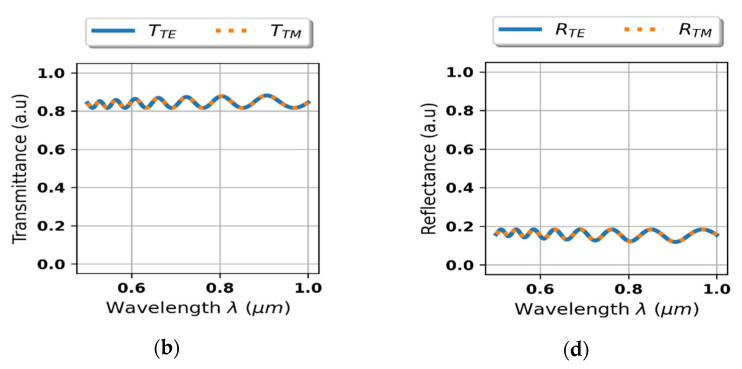
Transmittance (**a**,**b**) and reflectance (**c**,**d**) of the considered system with the HMM structure described via local (**b**,**d**) and non-local EMT approach (**a**,**c**).

Further analysis is focused on transmittance and reflectance of the considered system shown in the Figure 2. As we can see in the Figure 4a,b, the proposed device reveals strong rejection, i.e., no transmission, for light of arbitrary polarization for wavelengths shorter than approximately 0.625 μm. It is noteworthy that the transition between rejection and transmission range is accompanied with side lobes of oscillatory character, which is typical for edge and band optical filters [39]. Again, it is worth noting that this behavior cannot be predicted within the scope of local approximation, compare Figure 4a–d.

**Figure 5:**
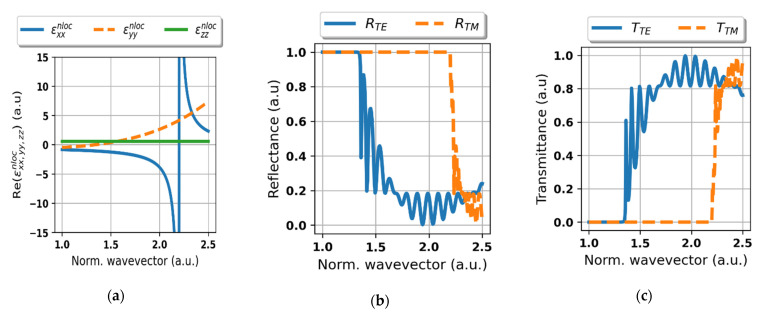
Effective permittivity tensor components (**a**), reflectance (**b**) and transmittance for TE and TM polarization (**c**) of the considered structure plotted vs. wavevector magnitude normalized to the wavevector of free-space wavelength *λ* = 600 nm at fixed angle of incidence *θ*_inc_ = 0° (air-side incidence).

In the Figure 5a–c, we present influence of the wavevector of incident radiation on effective dispersion of the considered HMM medium (Figure 5a) and reflectance/transmittance (RT) of the proposed system (Figure 5b,c) for a fixed wavelength *λ* = 600 nm and angle of incidence *θ*_inc_ = 0°. As we can see, the metallic-like behavior ($\varepsilon_{xx}^{nloc}<0,\varepsilon_{yy}^{nloc}<0$) is induced and sustained for waves of low wavevector magnitude, see Figure 5a. However, by increasing the magnitude of wavevector, it is possible to change its dispersion character from metal-like to dielectric ($\varepsilon_{xx}^{nloc}>0,\varepsilon_{yy}^{nloc}>0$) for both TE and TM polarization, see Figure 5a. These dispersion transitions, i.e., the change of sign of the permittivity tensor components, correspond to abrupt changes of reflectance/transmittance of the structure, see Figure 5b,c. Thus, we can distinguish three areas exhibiting different RT properties, namely: complete reflectivity for both polarizations (for |*k*| < 1.4), TE transmission-mode polarizer (1.4 < |*k*| < 2.2) and high transparency for light of arbitrary polarization (|*k*| > 2.2). Note that, the radiation impinging from air, i.e., |*k*| = 1, is completely blocked and reflected. Although waves of sufficiently high wavevector magnitude, i.e., |*k*| > 2.2 (for example provided by a high-index incident medium), are transmitted. This phenomenon provides a mechanism for inducing asymmetrical transmission for an arbitrary polarization of light by even a small change of wavevector’s magnitude. Thus, the change of wavevector’s magnitude provided by the assumed ZnSe substrate shall be sufficient to obtain the asymmetrical RT characteristics.

**Figure 6:**
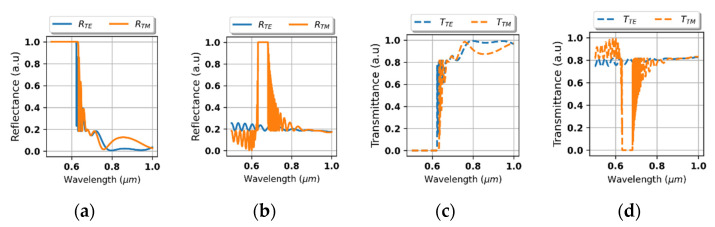
Spectral characteristics of reflectance (**a**,**b**) and transmittance (**c**,**d**) for waves impinging from the air side *θ*_inc_ = 0° (**a**,**c**) and from the substrate side *θ*_inc_ = 180° (**b**,**d**).

To verify the proposed mechanism for obtaining optical isolation, spectral characteristics of reflectance and transmittance of the considered system, i.e., the HMM structure deposited on ZnSe substrate, have been plotted for waves impinging from the air side, see Figure 6a,c, and the substrate side, see Figure 6b,d. As we can see, in the case of the air-side normal incidence, i.e., *θ*_inc_ = 0°, rejection for both polarizations, i.e., *R_TE_ = R_TM_* =1, can be observed for waves shorter than *λ*_0_ < 620 nm, see Figure 6a. Whilst in the case of the counter-directional propagation, i.e., normal incidence from the substrate-side (*θ*_inc_ = 180°), high transparency for an arbitrary polarization can be observed in the same spectral band, compare Figure 6c,d. The presented direction-dependent behavior of RT characteristics acts as a working principle of the proposed optical diode. It is worth to underline that this effect has been obtained for radiation of both TE and TM polarizations, which virtually constitutes all possible states of radiation in free-space and is essential to obtain performance of a “true” optical isolator. It is also noteworthy, that due to application of low-loss materials, the proposed structure operates in reflection-mode and does not introduce substantial insertion loss. What is more, for waves impinging from the substrate side the structure may also work as a TE polarizer, enabled by strong reflection for TM-polarized light of wavelengths between 0.62–0.7 μm, compare Figure 6a,b.

**Figure 7:**
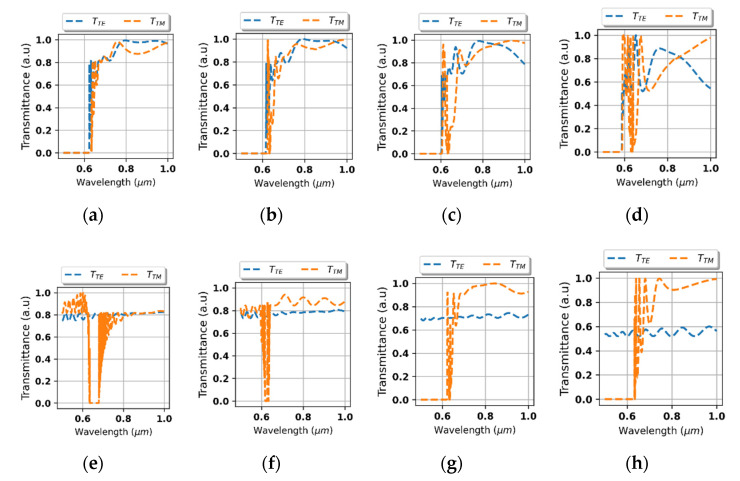
Spectral characteristics of transmittance for waves impinging from the air side (**a**–**d**) and substrate side (**e**–**h**) at various angles of incidence (**a**) 5°, (**b**) 25°, (**c**) 45°, (**d**) 65°, (**e**) 185°, (**f**) 205°, (**g**) 225°, and (**h**) 245°.

To further validate the fitness of the proposed system for optical isolation, it is required to determine whether the asymmetrical transmission is sustained for non-normal incidence. For this purpose, we have calculated transmittance spectra for waves impinging from the air and the substrate side at various angles of incidence, see Figure 7a–h. As we can see, the band of high reflection is well preserved for waves impinging from the air side even at angles of incidence up to approximately 65°, compare Figure 7a–d. In the case of substrate-side incidence, polarization-independent high transparency is sustained within a 155–205° cone, which correspond to 25°-deviation with respect to normal incidence. For larger angles, the transparency is retained for TE polarization only, see Figure 7e–g. However, since a part of possible states of radiation, i.e., TE polarization, is transmitted, the behavior of optical isolator is preserved.

**Figure 8:**
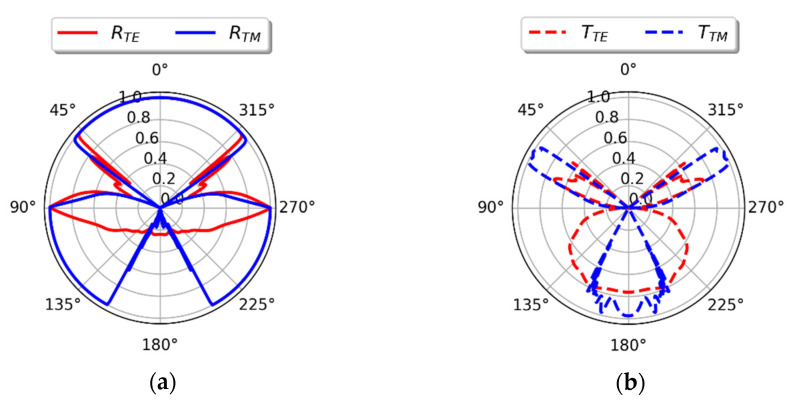
Reflectance (**a**) and transmittance (**b**) of the proposed optical diode plotted vs. angle of incidence at fixed wavelength λ_0_ = 600 nm.

To fully explore the sustainability of optical isolation over a wide range of angles, we have investigated 360°-angle radial characteristics of reflectance and transmittance for a wavelength arbitrarily chosen from the operating spectral range *λ*_0_ = 600 nm, see Figure 8a,b, where angles *θ*_inc_∊ (270°,90°) correspond to incidence from the air side while *θ*_inc_∊ (90°, 270°) are related to the substrate side incidence. It can be observed that the radiation of any polarization impinging from the air side is transmitted only within a narrow range of incidence angles, while waves incoming from the substrate side at an almost arbitrary angle of incidence are transported through the structure, see Figure 8a,b. Thus, the behavior of a “true” optical isolator is sustained within a wide-angle range. As indicated earlier, the structure may also serve as a TE polarizer for waves impinging from the substrate side at larger angles.

## 4. Conclusions

In this work, we have demonstrated for the first time that asymmetric transmission providing means for blocking backward propagation may be obtained in a grating-less planar structure without use of magnetic and non-linear effects. The demonstrated optical isolation is sustained over wide angle and relatively broad spectral range, which is of key importance in practical applications. Moreover, due to its simple planar geometry in comparison to the schemes proposed in References [25,26,27], which can be realized via well-established deposition techniques, the proposed optical diode may be easily fabricated onto almost any free-space optical element, such as a lens or a Brewster window, as well as embedded into an integrated optical system. It is worth to underline that similar non-local behavior, and consequently asymmetrical response, may be also obtained for different material composition, e.g., by substituting graphene with plasmonic material of low optical losses, and replicated for longer wavelengths by a proper design of the structure’s unit cell, as indicated in our previous work [28].

## Data Availability

All reported data and tools are available on request.
